# Research on Intelligent Wheelchair Attitude-Based Adjustment Method Based on Action Intention Recognition

**DOI:** 10.3390/mi14061265

**Published:** 2023-06-17

**Authors:** Jianwei Cui, Zizheng Huang, Xiang Li, Linwei Cui, Yucheng Shang, Liyan Tong

**Affiliations:** Institute of Instrument Science and Engineering, Southeast University, Nanjing 210096, China

**Keywords:** wheelchair posture adjustment, human intention recognition, deep learning

## Abstract

At present, research on intelligent wheelchairs mostly focuses on motion control, while research on attitude-based adjustment is relatively insufficient. The existing methods for adjusting wheelchair posture generally lack collaborative control and good human–machine collaboration. This article proposes an intelligent wheelchair posture-adjustment method based on action intention recognition by studying the relationship between the force changes on the contact surface between the human body and the wheelchair and the action intention. This method is applied to a multi-part adjustable electric wheelchair, which is equipped with multiple force sensors to collect pressure information from various parts of the passenger’s body. The upper level of the system converts the pressure data into the form of a pressure distribution map, extracts the shape features using the VIT deep learning model, identifies and classifies them, and ultimately identifies the action intentions of the passengers. Based on different action intentions, the electric actuator is controlled to adjust the wheelchair posture. After testing, this method can effectively collect the body pressure data of passengers, with an accuracy of over 95% for the three common intentions of lying down, sitting up, and standing up. The wheelchair can adjust its posture based on the recognition results. By adjusting the wheelchair posture through this method, users do not need to wear additional equipment and are less affected by the external environment. The target function can be achieved with simple learning, which has good human–machine collaboration and can solve the problem of some people having difficulty adjusting the wheelchair posture independently during wheelchair use.

## 1. Introduction

Wheelchairs, a commonly used medical rehabilitation device, can provide assistance for the travel and daily activities of the elderly and disabled. In the daily life of patients, it is often necessary to adjust the angle of the backrest and other parts of the wheelchair to meet their daily needs. For example, being in a lying position during rest is more conducive to body relaxation; sitting upright while eating is more beneficial for the swallowing function [1]; when sitting for a long time, it is necessary to adjust the angle of the wheelchair backrest in a timely manner to reduce the pressure on the buttocks and avoid pressure ulcers [2]; and some disabled athletes need to use wheelchairs to complete sports events, adjusting the posture of the wheelchair to change the position of the body’s center of gravity during use [3]. During the use of wheelchairs, the adjustment function of wheelchair posture and travel function are equally important.

During the use of electric wheelchairs by the elderly and some disabled individuals, it is difficult to accurately operate control panels in the form of buttons or screens due to factors such as decreased visual function and limited finger or upper-limb mobility. In addition, with the diversification of wheelchair functions, its control methods have become more complex, making it difficult for some groups, especially elderly users, to learn and master. For the hard problem of consciousness problem of operation in the use of wheelchairs, many scholars have studied and designed a variety of different types of human–computer interfaces to adapt for different types of disabled people [4,5] to improve the human–machine collaboration between humans and intelligent wheelchairs. Common human–machine interaction methods include speech recognition [6,7,8], head posture [9], EEG signals [10], electromyographic signals [11,12,13], and eye movement control [14]. However, the above human–computer interaction interfaces are mainly applied to the movement control of intelligent wheelchairs, and there is still a lack of adjustment methods that combine human–computer interactions in the adjustment function of wheelchair posture.

Regarding the method of adjusting the posture of a wheelchair, a multifunctional wheelchair was designed in reference [15] where the posture of the back and legs can be adjusted individually or in combination, and the adjustment mechanism of the wheelchair legs can also achieve the function of overcoming obstacles. In reference [16], the wheelchair seat was equipped with an adjustable threaded rod under it, which could adjust the posture of the seat by adjusting the height of the threaded rod. The wheelchair handle designed in reference [17] was equipped with a switch to control the hydraulic linkage, which could adjust the wheelchair posture by controlling the extension and retraction of the hydraulic linkage. The wheelchair used in reference [18] can change the angle of the backrest by manually adjusting the rotation axis at the backrest. The wheelchair designed in references [19,20] uses buttons to control the electric push rod on the wheelchair to achieve angle adjustment functions for the backrest, seat cushion, and other parts of the wheelchair, while reference [21] used touch-screen control. Based on the above literature, most existing wheelchair posture adjustment methods can only adjust the backrest, seat cushion, and pedals of the wheelchair separately, making it difficult to achieve collaborative control of multiple parts of the wheelchair. Therefore, during the adjustment process, it is easy to encounter situations where the wheelchair posture does not match the normal physiological posture of the human body; in addition, the posture adjustment function of standard electric wheelchairs lacks human–machine interaction and shared-control methods [22], often relying on the help of nursing staff, and lacks good human–machine collaboration during the use of wheelchairs, which to some extent limits the independent mobility of patients [23]. In our previous work [24], we developed an IoT intelligent wheelchair that can control the functions of wheelchair pedals and backrests through mobile apps. However, this process relies on manual control by the users and also lacks active perception and collaborative control for the users. This paper mainly conducts further research on methods for wheelchair posture adjustment.

According to analyses of the characteristics of people’s movements when using wheelchairs, when the user has the intention to stand, the contact force at the backrest of the wheelchair will significantly decrease, while the force at the pedal will increase. At the same time, the force area at the seat cushion will change (including the position of the force area and the size of the contact surface area). Due to the fact that people usually need to use wheelchair armrests to complete their up-movements, a downward force will be applied to the armrests. Based on these characteristics, this article proposes an intelligent wheelchair posture adjustment method based on action intention recognition, which judges the action intention by the change in contact force between the human body and the wheelchair.

Normally, the main contact areas between the human body and the wheelchair include the seat cushion, backrest, pedals, and armrests. For the first three areas, we installed array-type membrane pressure sensors to collect the force signals of the wheelchair, while at the armrests, we used four tension pressure sensors. The sensors at the seat cushion, backrest, pedal, and armrest correspond to the user’s buttocks, legs, back, feet, and arms, respectively. Through these force sensors, the wheelchair can fully perceive the changes in the user’s body force and identify potential intention information by identifying the changes in data. Due to the use of array sensors, the collected data matrix can be equivalent to gray image data, so this paper proposes a pressure image recognition algorithm based on VIT (Vision Transformer) for this part of data. At the same time, the VIT model has a higher efficiency in data utilization and can perform well even with a smaller dataset capacity. At present, the VIT model has a wide range of applications in image recognition tasks, such as object detection, image classification, and action recognition [25,26]. After practical testing, this method can effectively recognize and classify pressure images generated by different action intentions.

Compared with other intention recognition methods [27,28,29], obtaining intention information through body posture can better unleash user autonomy, making it easier for elderly people who may struggle to learn and operate them. At the same time, there is no need to wear additional equipment during use, which improves comfort and is more conducive to the health of skin tissue. In addition, due to the fact that the triggering of pressure signals is not affected by environmental factors such as sound and light, the system has stronger robustness and a wider range of applications. In summary, the system designed in this article is suitable for standard wheelchairs with various posture adjustments. Its operation is simple and easy to promote, and it is very user-friendly, especially for the elderly group. It has more practical significance in the application field of electric wheelchairs. The main contributions of this article are as follows:

- The system designed in this article deploys multiple force sensors on the upper part of the wheelchair, which provides a more comprehensive perception of human posture and is less affected by external environmental interference, enabling better recognition of human movement intentions;
- A pressure image classification model based on VIT is proposed, which converts pressure data into image form and classifies it to achieve recognition of action intention;
- This study also proposes an adaptive pressure-data acquisition method, which is applied to the pressure sensor acquisition card at the seat cushion to ensure good recognition performance for users of different weights.

The organizational structure of this article is as follows: In Section 2, the overall structure design and operation mode of the system are introduced. In Section 3, the data collection method and processing process are introduced. In Section 4, by analyzing the characteristics of two different types of sensor data, the corresponding data classification and recognition methods are provided. In Section 5, the feasibility of the intention recognition method and wheelchair posture collaborative adjustment method is verified through experiments. Section 6 provides conclusions and future prospects.

## 2. System Design

### 2.1. Electric Wheelchair Platform

**Figure 1:**
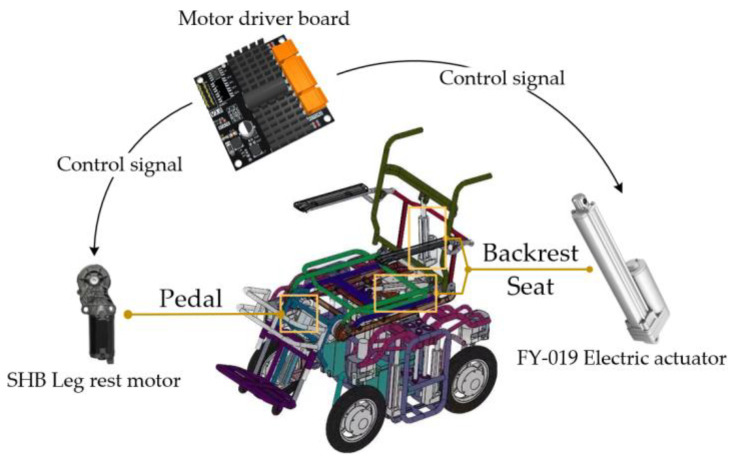
Actuator and installation position of electric wheelchair.

The system designed in this article is applied to an electric wheelchair, which is equipped with multiple adjustable electric actuators, mainly including two electric push rods and an angle adjustment motor. Through the control of the motor drive board, functions such as backrest rotation, pedal lifting, and seat cushion rotation can be achieved. The various actuating mechanisms and positions of the wheelchair are shown in Figure 1.

### 2.2. Selection and Installation of Sensors

There are two types of sensors used to detect force signals from the human body: array-distributed flexible film pressure sensors are used in the backrest, seat cushion, and footrest; and a tension pressure sensor is selected at the armrest.

**Figure 2:**
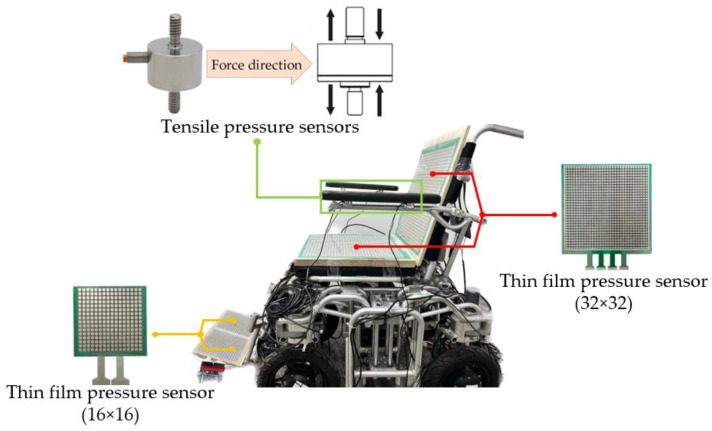
Diagram of sensor installation positions.

The array sensors installed at the backrest and seat cushion have 1024 independent sensing units, and the sensors at the foot pedal have 256 sensing units. Compared with other human pressure detection schemes [30,31,32,33,34], the thin film pressure sensor selected in this article has the advantages of large format, moderate sensing point size, and high resolution, as well as a wide range of applications in detecting the force distribution on the sole, seat cushion, and back. In addition, considering the behavior habits of people’s upper limbs when using wheelchairs, this article also installed four resistance strain-type tension and pressure sensors at the armrest of the wheelchair to sense the placement of the human arm. This resistance strain sensor is composed of elastomer, a resistance strain gauge, a compensation circuit, and other parts. When the elastomer is deformed by external tension or pressure, it will transfer the strain to the resistance strain gauge and change its resistance value and then convert it into a corresponding voltage signal or current signal through the circuit [35]. The installation positions of the two sensors on the wheelchair are shown in Figure 2.

### 2.3. Data Acquisition Software

**Figure 3:**
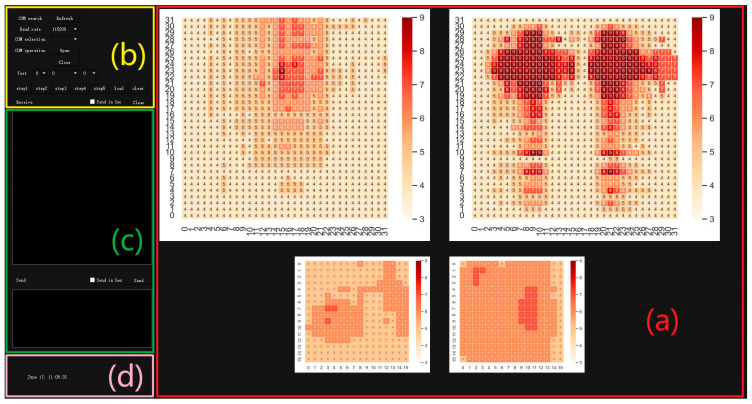
The operation interface of the system’s upper computer. (**a**): the display area of force distribution map, (**b**): serial port parameter setting area, (**c**): data transmission and display area, (**d**): time display area.

We have developed a system data collection software based on PyQt on the PyCharm platform. PyQt is a cross-platform toolkit for creating GUI (graphical user interface) applications, which can run on system platforms such as Windows, Linux, or Mac OS. It successfully integrates the Python programming language with the cross-platform Qt graphical user interface application framework and is currently one of the most powerful GUI libraries [36,37]. The running interface of the software is shown in Figure 3, and the functions of each region are as follows:

1. The display area of force distribution map: The collected data are converted into visual graphics using the heatmap function provided in the Seaborn library. Seaborn is a Python visualization library based on Matplotlib, which allows for a more intuitive view of pressure distribution;
2. Serial port parameter setting area: The parameters that can be set for the serial port include the baud rate and serial port number. Other default parameters include the stop bit, check bit, and data bit as 1, NONE, and 8, respectively;
3. Data transmission and display area: The upper half is the data-receiving display area, and the lower half is the data-sending display area, both of which support HEX mode;
4. Time display area: Used to display the date when the upper computer is running.

## 3. Data Acquisition and Processing

### 3.1. Dataset

The dataset used in this article was collected from 20 volunteers, whose height and weight ranges were recorded. Each volunteer sat in a wheelchair according to their personal habits and repeated four movements, including sitting upright, lying down, getting up, and standing, to collect 50 pieces of data each (with the wheelchair lying flat when getting up). Each piece of data included the pressure data at the armrests, seat cushions, backrest, and pedals.

### 3.2. Data Acquisition

**Figure 4:**
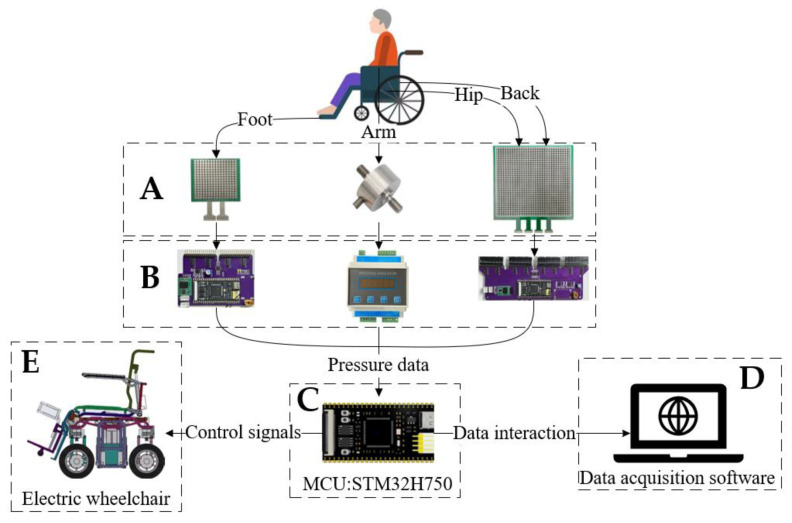
System overall structure diagram: (**A**) force sensors, (**B**) capture cards, (**C**) MCU, (**D**) data acquisition software, and (**E**) electric wheelchair.

The overall structure of the system is shown in Figure 4. We collected pressure information from the human body through sensors (A) and supporting data acquisition cards (B). The collection card of the thin film pressure sensor was designed by us, and each collection card comes with an MCU for preprocessing the pressure information. Through this distributed architecture, the collection rate of the system can be greatly accelerated. The collection card of the tension pressure sensor adopts the HYDG-BS rail-type transmitter produced by China Bengbu Hengyuan Sensor Technology Co., Ltd. (Bengbu, China) (which was calibrated before use). All data acquisition cards are connected to the RS485 bus of the system. The data acquisition, storage, and visualization operations are completed through the data acquisition software introduced in Section 2.3. The interaction between the acquisition software and the 485 bus is completed by the overall MCU (C). In addition, the control functions of each linear actuator of the wheelchair are also completed by the MCU (C).

### 3.3. Adaptive Pressure-Data Acquisition Method

In the actual testing process, we noticed that the seat cushion is the main force bearing part of the wheelchair and the main data source for intention recognition. However, different body types of passengers can produce different pressure effects. Therefore, we propose an adaptive pressure-data collection method. This method aims to ensure that passengers of different weights have pressure data with relatively uniform characteristics, reducing recognition errors caused by weight differences among users.

This function acts on the data acquisition card of the thin film pressure sensor at the seat cushion. The working principle of this data acquisition card is as follows: the core working circuit of the acquisition card is a voltage series negative-feedback amplification circuit, with the input voltage $U_{i}$ coming from the DAC pin of the MCU and the output voltage $U_{o}$ connected to the reverse input terminal of the operational amplifier through the reverse feed resistance $R_{f}$. The feedback resistor $R_{f}$ is a fixed value resistor with a resistance value of 100 kΩ, and the resistance R represents the sensing unit on the pressure sensor. The sensing unit is essentially a varistor, and its characteristic curve is shown in the following figure. Finally, the pressure on the sensing unit is determined based on the output voltage $U_{o}$ collected by the ADC pin of the MCU.

**Figure 5:**
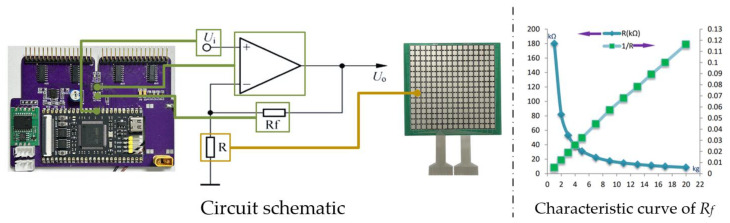
Circuit diagram of a voltage series negative-feedback amplifier.

According to the characteristics of the series negative-feedback amplification circuit, the output voltage in Figure 5 can be expressed as:

(1) $$U_{o}=(1+\frac{R_{f}}{R})\times V_{-}=(1+\frac{R_{f}}{R})\times U_{i}$$

From Equation (1), it can be seen that when $U_{i}$ is a constant value, if the weight of the passenger is relatively light, the resistance value of resistance R only slightly decreases due to the small pressure, and the range of the output voltage $U_{o}$ change is small; on the contrary, a person with a larger weight sitting in a wheelchair will cause a significant decrease in the resistance value of resistance R, which will result in a large number of sensing units reaching the upper limit value of the measurement. Therefore, the acquisition card needs to adaptively adjust the output voltage $U_{i}$ value before starting the measurement to ensure that the subsequently collected pressure values are always within a relatively uniform range. In the process of data collection, we define the point where the pressure value reaches its maximum value as the full pressure point, and its quantity is represented by M. The proportion P of the full pressure point of the sensor can be expressed as:

(2) $$P=\frac{M}{1024}$$

The method designed in this section adjusts the output voltage $U_{i}$ by detecting the proportion of the full pressure point. The output voltage $U_{i}$ can be expressed as:

(3) $$U_{i}=U_{i0}\times \left[1+\frac{M_{0}-M}{1024}\right]$$

In the formula, $U_{i0}$ is the initial voltage, and $M_{0}$ is the default number of full pressure points in the system. After one or more adjustments, when $P\in \left(0.15,0.25\right)$ is reached, the algorithm adjustment is considered complete. The current output voltage $U_{i}$ is recorded and set, and subsequent data from this frame are considered valid.

### 3.4. Pressure Data Visualization Processing

**Figure 6:**
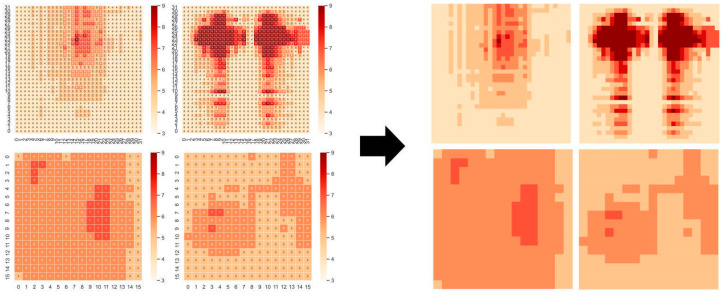
Raw pressure data images and converted images.

The pressure data form of the thin film pressure sensor is 32 × 32 (or 16 × 16). The data arrangement characteristics of the matrix are similar to the image data, with a frame of 32 × 32. The pressure data of 32 can be seen as a graph composed of 32 × 32. A picture composed of 32 pixels can be converted into image format for this form of data. We use the upper computer in Section 2.3 to visualize the collected pressure data, where different colored blocks represent sensing units with different pressure values. This transforms the classification problem of pressure data into an image classification problem. To facilitate subsequent recognition, the image is converted to 224 × 224. The images before and after processing are shown in Figure 6.

## 4. Methods

In the system studied in this article, there are two types of data generated by human action intentions: one is pressure images from thin film pressure sensors, and the other is tension pressure sensor data from the armrest. Therefore, the method in this article is mainly divided into two parts: (1) Classifying pressure images under different human intentions using the VIT algorithm; (2) Serialization and classification method for armrest pressure data.

### 4.1. VIT-Based Pressure Image Recognition Algorithm

#### 4.1.1. Pressure Image Input Processing

The Transformer model was originally used in the field of natural language processing, mainly dealing with two-dimensional sequence data such as text, sentences, and paragraphs. Therefore, before using the Transformer model to recognize pressure images, the first step is to reduce the dimensionality of the three-dimensional image data and convert it into a two-dimensional sequence shaped like $\left(N,D\right)$ (N represents the length of the sequence, D is the dimension of the vector).

For a pressure image $x\in R^{H\times W\times C}$ (H and W represent the height and width of the image, C represents the number of image channels), first divide it into several fixed-size *P* image blocks, each of which can be represented as $x_{p}\in R^{N(P^{2}C)}$. Flatten these image blocks to obtain a sequence of image blocks with a length of N.

(4) $$N=HW/P^{2}$$

**Figure 7:**
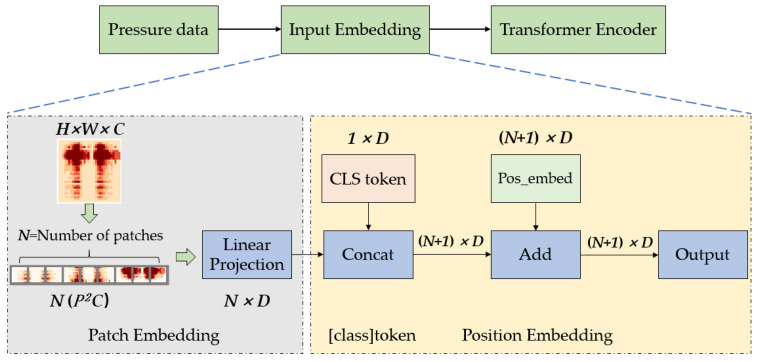
Image data conversion process.

At this point, the dimensions of the image block are $\left(P^{2}\times C\right)$, and then it is transformed into D through linear mapping to obtain a two-dimensional sequence $\left(N,D\right)$ containing image information. Then, insert a class token for classification before the image sequence with dimensions consistent with the image sequence, and the image sequence becomes $\left(N+1,D\right)$. Finally, a position embedding is added to the image sequence, which preserves the original position information of the image block and prevents the loss of position information in subsequent recognition processes. After adding position encoding, the dimensions of the image sequence remain $\left(N+1,D\right)$. The above process is the entire process of pressure image preprocessing, which essentially involves dimensionality reduction of the image data. The sequence $\left(N+1,D\right)$ obtained through the above operations is the input data of the Transformer model, and the above process is called input embedding, as shown in Figure 7.

#### 4.1.2. Feature Extraction Method

The extraction of data features in Transformer Encoder is mainly achieved through the multi-head attention module, which is composed of multiple self-attention modules. When extracting features from a single self-attention module, the two-dimensional sequence containing pressure image information after input embedding is first divided into multiple one-dimensional subsequence components *x*.

(5) $$X=\left\{x_{1},x_{1},x_{1},\ldots ,x_{N+1}\right\}$$

(6) $$x_{i}=\left(i,D\right)$$

Each component $x_{i}$ is linearly transformed through three matrices $W^{Q},W^{K},W^{V}$ (randomly initialized and updated with training parameters) to obtain three vectors $q^{i},k^{i},v^{i}$.

(7) $$q^{i},k^{i},v^{i}=x_{i}W^{Q},x_{i}W^{K},x_{i}W^{V}$$

Here, q represents query and k represents key. Next, perform the weighted inner product operation on all vectors q and k, respectively, to obtain the value $\alpha_{i,j}$, which is essentially to match how approximate the information of these two vectors is.

(8) $$\alpha_{i,j}=\frac{q^{i}k^{j}}{\sqrt{d_{k}}}$$

In the equation, $\sqrt{d_{k}}$ is the square root of the dimension of the k vector, and it is divided by this value to maintain the stability of the gradient value in subsequent training. Then, perform the Softmax operation on $\alpha_{i,j}$ and multiply it with vector $v^{i}$ to obtain the final feature vector $b^{i}$.

(9) $$Softmax\left(z_{i}\right)=\frac{e^{z_{i}}}{\sum_{c=1}^{C}e^{z_{c}}}$$

(10) $$Attention\left(Q,K,V\right)=Softmax\left(\frac{QK}{\sqrt{d_{k}}}\right)V$$

**Figure 8:**
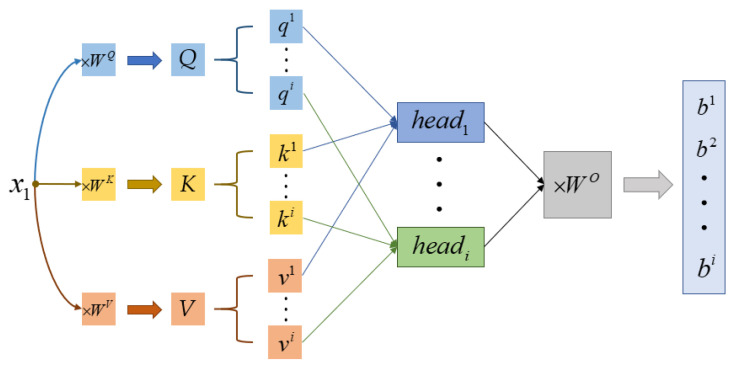
Feature extraction process of multi-head attention.

The essence of the Softmax function is to sum all the values in $\alpha_{i,j}$ to 1. The entire feature extraction method can be represented by the following formula, where it is the case of a single self-attention module. The multi-head attention module divides each group of $q^{i},k^{i},v^{i}$ into multiple components based on the number of heads for self-attention operation then finally concatenates all the obtained results $b^{i}$ to obtain the output result, as shown in Figure 8.

(11) $$MultiHead\left(Q,K,V\right)=Concat(head_{1},\ldots ,head_{h})W^{O}$$

(12) $$head_{i}=Attention(QW_{i}^{Q},KW_{i}^{K},VW_{i}^{V})$$

#### 4.1.3. Stress Image Classification and Intent Recognition

After extracting features from the image sequence data through the Transformer Encoder, we extract the CLS token classification vector (dimension $\left(1,D\right)$) and input it into the fully connected layer for pressure image classification. The entire classification function is implemented by the MLP head layer. The classification results of each part are weighted according to a certain weight to obtain the final classification result $P_{\alpha }$, which can be expressed as:

(13) $$P_{\alpha }=\sum_{i=1}^{4}\mu_{i}P_{i}$$

**Figure 9:**
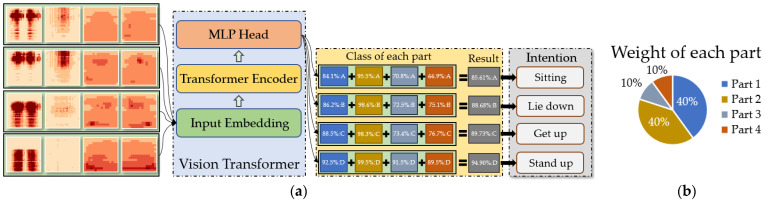
VIT model identification process: (**a**) data transfer process; (**b**) weight of each part, A–D represents four different intentions.

When the probability $P_{\alpha }$ reaches 85%, it is considered as a valid classification, and then the preliminary human intention can be identified according to the target category. The data transmission and recognition during the entire algorithm operation process are shown in Figure 9.

### 4.2. Handrail Pressure Data Serialization Classification Method

According to the working characteristics of the pull-pressure sensor at the armrest, the sensor will output a negative value when under pressure and a positive value when under tension. The higher the absolute value, the greater the force. We will divide dataset D in Section 3.1 into four sub datasets, $D_{1},D_{2},D_{3},D_{4}$ according to intention, then convert the data from the four sensors into vector form in a fixed order.

(14) $$D_{i}=\left\{s_{1}^{i},s_{2}^{i},\ldots ,s_{n}^{i}\right\}$$

Perform averaging operations on the data of each sub-dataset to obtain four benchmark sequences $S_{0}^{1},S_{0}^{2},S_{0}^{3},S_{0}^{4}$.

(15) $$S_{0}^{i}=\frac{\sum_{j=1}^{n}s_{j}^{i}}{n}$$

**Figure 10:**
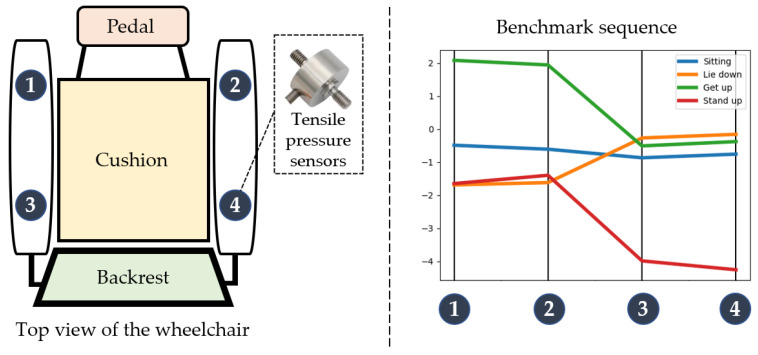
Sensor placement order and benchmark sequence, 1–4 represents sensors installed in different positions.

Each reference sequence contains four elements, representing the values of four pressure sensors. The arrangement order of the four sensors and the corresponding reference sequence with different intentions are shown in the following Figure 10.

For an unknown sequence collected, we use Euclidean similarity to determine the degree of similarity between the unknown sequence and the reference sequence in order to determine which situation the sequence is most likely to belong to. Euclidean similarity can be expressed using the following formula.

(16) $$E(p,q)=\sqrt{\sum_{i=1}^{n}{\left(p_{i}-q_{i}\right)}^{2}}$$

**Figure 11:**
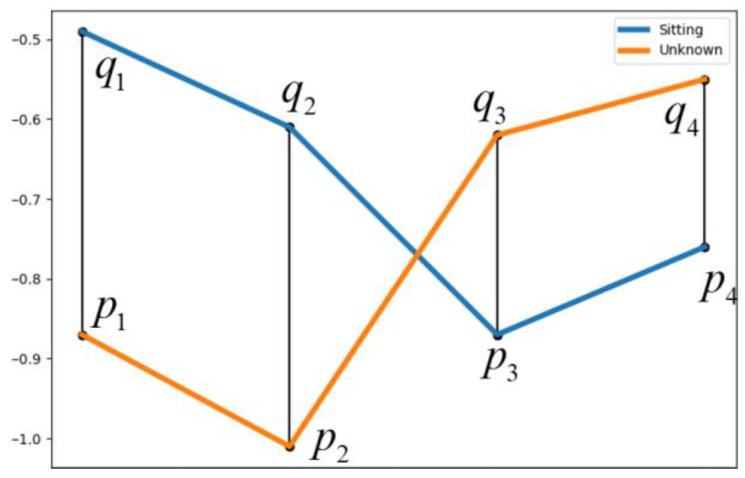
Diagram of similarity between sequences.

In the equation, E represents distance, and the elements p and q come from unknown sequences and reference sequences, respectively, and the calculation process is shown in Figure 11.

By comparing the similarity between the unknown sequence and the four benchmark sequences, the smaller the distance E is, the closer the unknown sequence is to a certain benchmark sequence. The probability of $P_{\beta }$ belonging to this sequence can be expressed as:

(17) $$P_{\beta }=\left(N-E_{\min}\right)/N$$

In the formula, N is the similarity threshold, set according to the measurement range of the sensor; and $E_{\min}$ is the minimum of the four distance values. Finally, determine whether the unknown sequence belongs to a certain action intention based on the probability $P_{\beta }$.

Based on the intention recognition situation in Section 4.1, when both $P_{\alpha }$ and $P_{\beta }$ are greater than 85%, it is determined that the human body has intention to act. When the $P_{\alpha }$ is greater than 85% and no pressure is detected at the armrest, it is also determined that the human body has intention to move. In other cases, it is believed that no obvious action intention has been identified. In addition, in the case of potential motion intention recognition errors, if the user can change their body’s center of gravity, the system’s safety stop can be triggered; that is, they only need to slightly lean to transfer the center of gravity to the side of the wheelchair. When the system detects that the human body’s sitting posture is not correct, all linear actuators will stop operation and restore the initial state. Throughout the use of the wheelchair, the priority of the safety function is higher than that of the intention recognition function.

## 5. Experiments and Results

This section verifies three aspects through experiments: (1) Adaptive pressure-data collection; (2) The accuracy of human action intention recognition; (3) The collaborative adjustment function for chair posture.

### 5.1. Performance Test of Adaptive Pressure-Data Collection Algorithm

**Figure 12:**
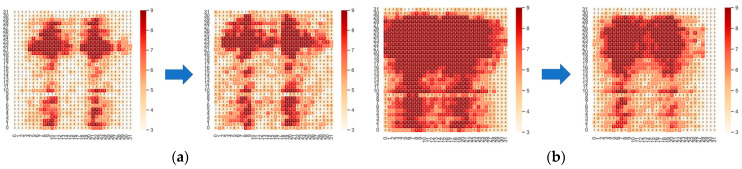
Adaptive pressure-data acquisition algorithm effect diagram: (**a**) results for 54 kg subject; (**b**) results for 78 kg subject.

The default parameter configuration of the system is based on a weight of 65 kg, where the $U_{i0}$ size is 1.5 V and $M_{0}$ is 200. Two volunteers weighing 54 kg and 78 kg were used to test for the effectiveness of the adaptive pressure-data collection, as shown in Figure 12.

The point with a pressure value of 9 in the figure is the full pressure point ($value\in \left[3,9\right]$). The number of full pressure points M and the proportion of full pressure points P before and after adjustment are shown in Table 1. The adjusted proportion of full pressure points $P\in \left(0.15,0.25\right)$ meets the expected effect.

### 5.2. Human Movement Intention Recognition Effect Test

In the actual test, the pressure image recognition algorithm uses the VIT-Base/16 model. We divide the dataset in Section 3.1 into a training set and verification set according to the ratio of 9:1 and train the network parameters using the transfer learning method.

**Figure 13:**
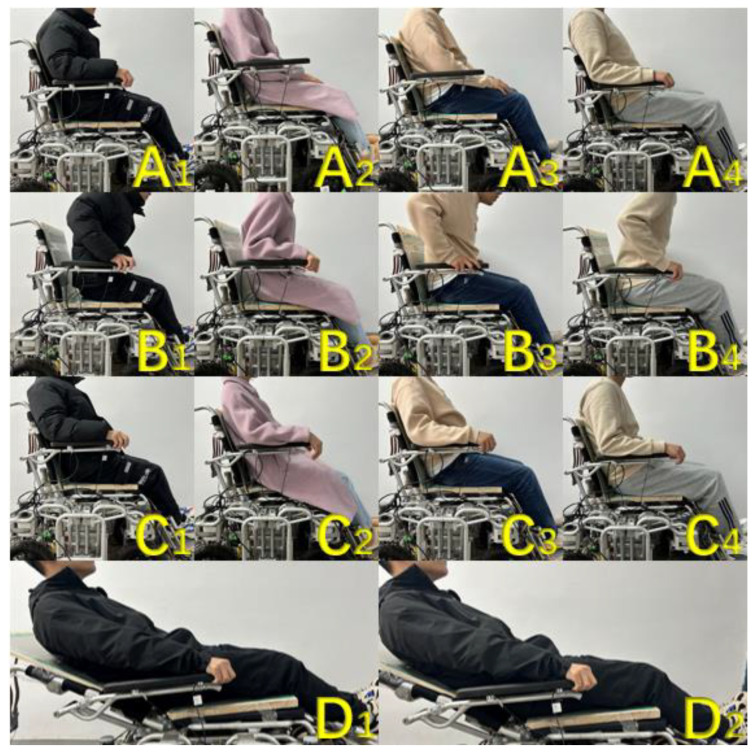
Human movement intention recognition experiment, (**A**): sitting, (**B**): standing up, (**C**): lying down, (**D**): sitting up.

The formal experimental session consisted of twenty participants, including fourteen males and six females, with a range of height, body mass, and weight scores ranging from 165 cm to 178 cm and 58 kg to 86 kg. The experimental process was as follows:

- Before the experiment began, each participant was naturally sitting in a wheelchair, with their body and limbs in free contact with various parts of the wheelchair. The system used the method described in Section 3.3 to adjust the pressure data at the seat cushion. As this part of the experiment only tested the accuracy of intention recognition, in order to maintain consistency in the experiment, the adjustment function of the wheelchair was turned off during the testing process;
- After the adjustment was completed, the formal experimental phase began. Each participant was free to make any number of attempts to lie down, stand up, and stand up within a 5-min time frame. The experimental process is shown in Figure 13, where A1~A4 represent the normal sitting posture, B1~B4 represent the state when the human body has the intention to stand up, C1~C4 represents the state when the human body has the intention to lie down, and D1~D2 represents the state when the human body has the intention to sit up;
- The number of occurrences of actual action intentions during the experiment was recorded by on-site researchers. The system will automatically detect the human body’s action intentions and record the number of occurrences. Except for the three types of action intentions mentioned above, the number of occurrences is not counted. Finally, the accuracy of recognition is obtained by dividing the number of intentions detected by the system by the actual number of action intentions (the detection frequency of the system is 2 Hz).

The data recorded throughout the entire experimental process are shown in Table 2, with the accuracy rates of intention recognition for lying down, getting up, and standing being 95.12%, 98.7%, and 100%, respectively.

### 5.3. Wheelchair Posture Adjustment Function Test

**Figure 14:**
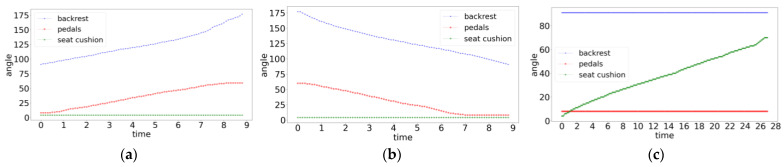
Changes in the angles of various parts of the wheelchair during posture adjustment: (**a**) lying down; (**b**) sitting up; and (**c**) standing up.

**Figure 15:**
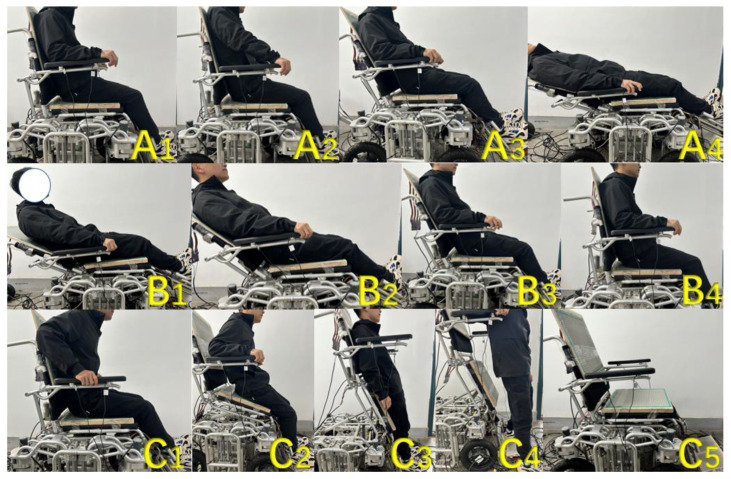
The effect of wheelchair posture adjustment function, (**A**): lie down, (**B**): sit up, (**C**): stand up.

This section mainly tests the wheelchair posture adjustment function. Before starting the test, the wheelchair was first reset, and all parts were adjusted to the angle when sitting upright. Next, the wheelchair posture adjustment functions corresponding to the intentions of lying down, getting up, and standing were tested. The posture changes of the wheelchair during the experiment are shown in Figure 14:

- Part A in the figure shows the posture adjustment process of the wheelchair when the passenger has a lie-down intention while sitting upright. When the system detects that the user has a lie-down intention while sitting upright (Figure 15(A1)), the push rod at the backrest of the wheelchair will contract to adjust the angle of the backrest in a counterclockwise direction. The angle adjustment motor at the pedal will also synchronously rotate counterclockwise to lift the pedal, and the two electric actuators can achieve collaborative control throughout the entire process to ensure that the human body always maintains a comfortable and reasonable posture (Figure 15(A2–A4));
- Part B in the figure shows the posture adjustment process of the wheelchair when the passenger intends to sit up while lying down. When the system detects that the user in a lying state has an intention to sit up (Figure 15(B1)), the electric push rod at the backrest of the wheelchair will extend to adjust the backrest in a clockwise direction, and the angle adjustment motor at the pedal will synchronously rotate clockwise to lift the pedal. Throughout the entire process, the backrest and pedals of the wheelchair will be in a synchronized adjustment state (Figure 15(B2–B4));
- The Figure 15(C1–C5) parts in the figure show the posture adjustment process of the wheelchair when the passenger intends to stand up while lying down. When the system detects that the user is in a sitting position and has an intention to stand up (Figure 15(C1)), the push rod under the seat cushion will extend to support the cushion part. At the same time, the angle adjustment motor at the pedal will rotate clockwise to meet the standing needs and the above two functions will be performed simultaneously (Figure 15(C2,C3)). After completing the auxiliary standing function, the person can leave the wheelchair (Figure 15(C4)). When it is detected that the person has left the wheelchair, the wheelchair will automatically reset to its initial state (Figure 15(C5)).

## 6. Conclusions and Prospects

In this study, we propose an intelligent wheelchair posture adjustment method based on action intention recognition. Firstly, the system takes pressure signals from various parts of the human body as inputs and can assist in controlling the wheelchair by identifying features with different intentions. Compared with traditional control methods, this system breaks free from the constraints of manual joystick- and button-control methods and improves the synergy in the wheelchair attitude-based adjustment process. Due to the fact that the pressure signal comes from human contact, it is less affected by the external environment compared to technologies such as speech recognition. At the same time, there is no need to wear any equipment during use, providing higher comfort. Users only need to control the wheelchair according to their usage habits, making it easy to operate and master.

In the design process of the system, we designed a data acquisition card equipped with MCU for several main array thin film pressure sensors, which can independently collect and process pressure data, improving the overall performance and operational efficiency of the system. In order to adapt the system to a wider audience, we added an adaptive pressure-data collection algorithm to the data collection card. This algorithm can adjust the cushion pressure data of users with different weights, providing distinctive input data for subsequent recognition functions. We also proposed using the VIT deep learning classification model to identify pressure distribution images. By visualizing the pressure data, the recognition of pressure data is combined with image classification tasks. After experimental testing, the recognition accuracy of each intention is above 95%. When the intention is recognized, the wheelchair makes corresponding adjustments, and the system operates stably within a certain time range.

In response to the poor performance of some intention recognition, we plan to consider expanding the system’s dataset and training the model by collecting more user position data to improve the recognition rate of the model. In addition, we are also considering combining intention recognition with the operation of wheelchairs, developing more types of intention recognition and corresponding functions, and improving the human–machine interaction experience of intelligent wheelchairs.

## Figures and Tables

**Table 1:** Adaptive pressure-data collection algorithm test data table.

Weight	Data Status	M	P
54 kg	Unadjusted	99	0.0966
	Adjusted	158	0.1542
78 kg	Unadjusted	473	0.4619
	Adjusted	204	0.1992

**Table 2:** Intent recognition experiment data sheet.

Intentions	Occurrence Times	Recognition Times	Accuracy Rates
Lie down	82	78	95.12%
Get up	77	76	98.7%
Stand up	79	79	100%

## Data Availability

Not applicable.
